# New birthweight percentiles by sex and gestational age in Southern China and its comparison with the INTERGROWTH-21st Standard

**DOI:** 10.1038/s41598-018-25744-7

**Published:** 2018-05-15

**Authors:** Fei Yao, Huazhang Miao, Bing Li, Yuntao Wu, Qingguo Zhao

**Affiliations:** 1 Department of Healthcare, Guangdong Women and Children Hospital, No. 521 Xingnan Road, Guangzhou, 511442 Guangdong China; 2 Epidemiological Research Office of Key Laboratory of Male Reproduction and Genetics (National Health and Family Planning Commission), Family Planning Research Institute of Guangdong Province, No. 17 Meidong Road, Guangzhou, 510600 Guangdong China

## Abstract

We first showed the sex- and gestational age (week)-specific birthweight distributions from primiparous and multiparous mothers. Birthweight percentiles were created by using the Lambda Mu Sigma (LMS) method. We established the birthweight percentiles in Southern China and provide clinicians and researchers with up-to-date population norms of birthweight percentiles. Then we use the data from Birth Certificate System between Jan 1st, 2017 and Oct 31st, 2017, which included 1,245,364 live births to calculate SGA and LGA ratio by these three birthweight references- the new INTERGROWTH-21st standard, China’s 2015 research standard and our birthweight percentiles. The overall prevalence of SGA estimated by our standards, the China’s 2015 research standards and INTERGROWTH-21st standards, were 10.21%, 12.93% and 7.98%, respectively, whereas the overall prevalence of LGA was 9.88%, 4.48% and 8.37%, respectively.

## Introduction

Neonatal birthweight has been an important indicator of common concern in obstetrics and neonatology. This factor is critical for the study of adverse pregnancy outcomes and the prediction of neonatal mortality and morbidity. Extreme (large or small) neonatal birthweight increase the risks of diseases in adulthood^1,2^. The birthweight percentile is used to monitor the clinical measurement within the population value of the individual within the range. Infants who are SGA or LGA, are commonly defined as below the 10th or above the 90th centiles according to the birthweight percentile reference standard^3,4^. Brazil, India, Korea, Canada, United States, Japan, and Australia have published studies for assessing birthweight for gestational age (week)^5–11^. Nowadays, INTERGROWTH-21st reported a new global newborn birthweight standard, and attempted to define an international birthweight standard in the context of optimal maternal health and fetal growth^12^.

In our analysis, we first showed the sex- and gestational age (week)–specific birthweight distributions for primiparous and multiparous mothers separately. Using the birth certificate data in 2017, the SGA ratio and the LGA ratio obtained through the standards’ 10th and 90th percentile values. Standards included the new INTERGROWTH-21st standard and China’s 2015 research standard then we compare the ratios. In China, the Chinese newborn collaboration network also produced a newborn birthweight standard in 2015^13^. Since birthweight may differ by race and ethnicity, and there have been increasing number of older and multiparous mothers after the general implementation of two-child policy in China since 2016, the birthweight standard may change and the outdated classification of infants as SGA or LGA may not be accurately defined. This paper is to establish our birthweight standard in Guangdong province in Southern China and study up-to-date birthweight changes and abnormal birthweight risk factors.

## Materials and Methods

All birth data were obtained from the Guangdong Provincial Birth Certificate System, about 1900 medical institutions, which collected all information about infants who were admitted to medical institutions accredited to midwifery in Guangdong Province. After the birth, maternity medical workers would bare the baby on the electronic scales, read the stable weight data, weighing accuracy is 1 g; on the other hand, health care attendants or midwives would fill in the newborns’ delivery information in the regional maternal and child information system. The system setting logic was corrected to ensure the correct range. At last, regional maternal and child information would upload to Guangdong Provincial Birth Certificate System. The Chief of Midwifes and the Chief of Physicians in hospitals confirmed the information after data entry. Before the birth certificate is issued, the Department of Medical Administration and parents also confirmed the birth information. All the information was verified by the medical professional. The birth registry database maintains the date of birth, gestational age (week) at birth, birthweight, infant sex, parents’ ages, registered residence and parity. We included the babies in the study which were born except for stillbirth, death within seven days, and birth defects. Our analyses were based on 5,516,173 births after exclusions. The study was reviewed and approved by Ethics Committee of Guangdong Women and Children Hospital.

The raw data of all newborns (1,771,534 in 2014, 1,627,254 in 2015, and 1,757,385 in 2016) were analyzed. The gestational age (week) was measured combining with mother-reported last menstrual period, early pregnancy ultrasound, and postnatal gestational age (week) assessment. It was expressed in completed weeks. The birthweight was measured immediately after the delivery with measurement accurate to 1 gram. Birthweight percentiles were created by using the Lambda Mu Sigma (LMS) method, which can be fitted within the GAMLSS package by assuming that the birthweight has a Box-Cox Cole and Green (BCCG) distribution^14,15^. The smoothed data were represented by the BW percentile curves. The curves appeared at intervals of one week by gestation and separated into all single births. We also constructed separate curves and tables for male and female newborns for the 3rd, 5th, 10th, 25th, 50th, 75th, 90th, 95th, and 97th percentiles from 25 to 42 completed weeks based on smoothed estimated curves. The charts and tables were stratified by gender and parity for the 3rd, 5th, 10th, 50th, 90th, 95th and 97th percentiles, and SD were obtained after smoothing. SGA was defined as weight below the 10th percentile of a sex- and gestational age (week)–specific birthweight curve.

Then we use the data from Birth Certificate System between Jan 1^st^, 2017 and Oct 31^st^, 2017, which included 1,245,364 live births to calculate SGA and LGA ratio by three birthweight references. The 10th percentile values of the above three birthweight references were compared against each other, and the difference in cutoff weight at each gestational age (week) was calculated. The same comparison was performed for the 90th percentile values. The GAMLSS package (version 5.0.6) for R statistical software (version 3.4.2) was used for the analysis.

## Results

A retrospective survey for data pertaining to birthweight, maternal parity and infant gender was conducted on 5,516,173 singleton live births at gestational age (week)s 25–42 weeks from January 1^st^, 2014 to December 31^st^, 2016.We included the babies in the study which were born except for stillbirth, death within seven days, and birth defects. After exclusion of birthweight outliers, we included 5,516,173 single neonates in the analysis, comprising 710,480 firstborn boys, 649,882 firstborn girls, 2,053,993 later-born boys, and 1,741,818 later-born girls. Males comprised 53.61% of the births, and 25.70% of infants were firstborn. Late premature infants born at 34–36 weeks’ gestation and premature infants born at <34 weeks’ gestation accounted for 3.64% and 0.78% of the total infant population, respectively. 20 to 34 years-old mothers accounted for 84.10% of all pregnant women, among which 8.90% were above 35 years-old, and was 18.20% of fathers were older than 35 years-old. Vaginal delivery and cesarean section delivery accounted for 72.10% and 27.70% respectively, while the remaining delivery modes were unclear.

**Figure 1:**
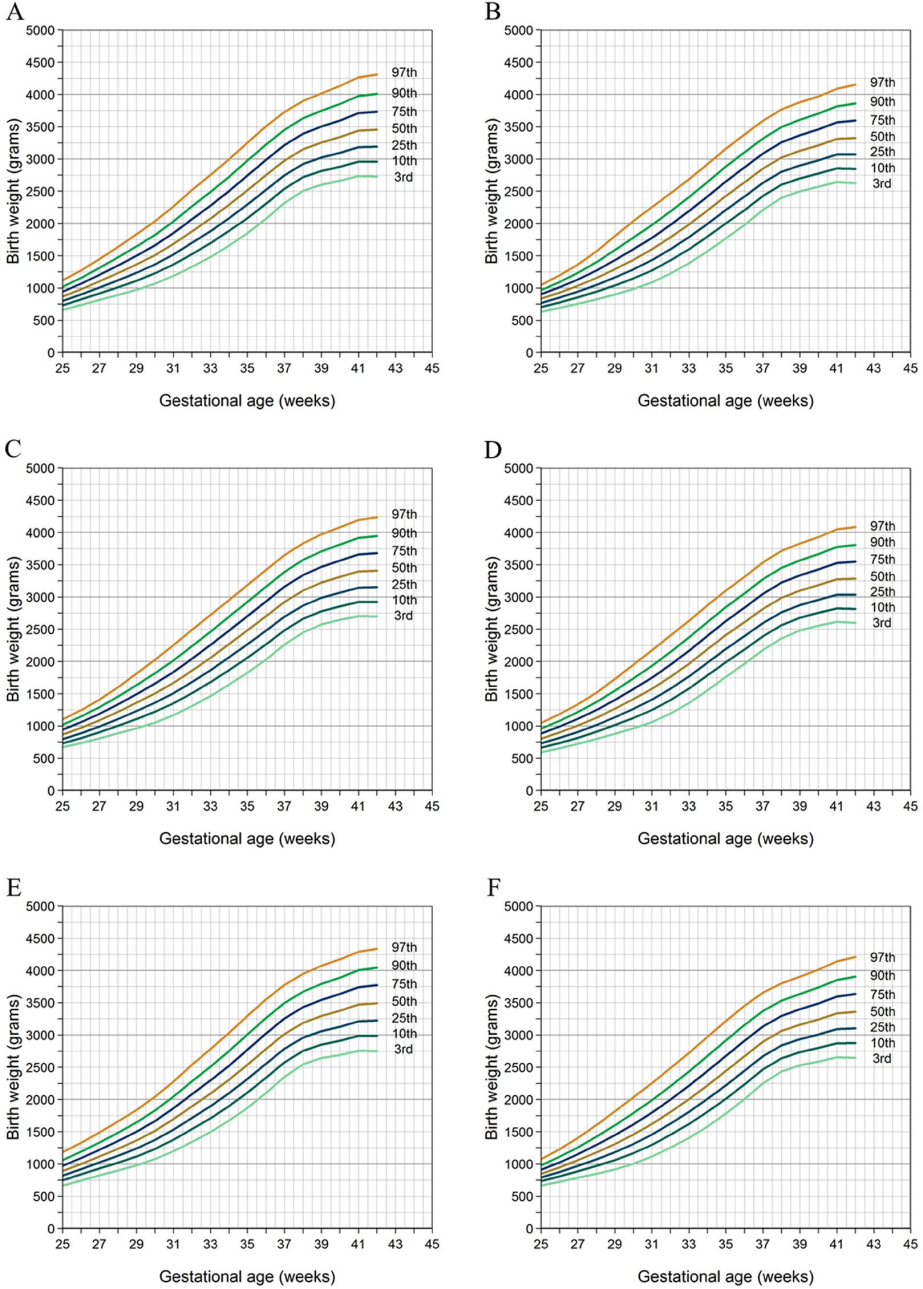
Smoothed percentiles for weight (gms) by gestational age (week) (weeks) for (**A**) male and (**B**) female babies. Smoothed percentiles for weight (gms) by gestational age (week) (weeks) for (**C**) male and (**D**) female babies from primiparous mothers. Smoothed percentiles for weight (gms) by gestational age (week) (weeks) for (**E**) male and (**F**) female babies from multiparous mothers. (**A**) We first showed the male and gestational age (week)-specific birthweight distributions from mothers who may had ever delivered or did not give birth ever. Curve fitting of the third percentile birthweight of each gestational age (week) (25–42 gestational age (week)) was presented. Above the third percentile, it is the 10th percentile, the 25th percentile, the 50th percentile, the 75th percentile, the 90th percentile, and the 97th percentile of each gestational age (week). (**B**) Showed the female and gestational age (week)-specific birthweight distributions from mothers who may give birth once or not. (**C**) Representative the male and gestational age (week)-specific birthweight distributions from primiparous mothers. (**D**) Representative the female and gestational age (week)-specific birthweight distributions from primiparous mothers. (**E**) Representative the male and gestational age (week)-specific birthweight distributions from multiparous mothers. (**F**) Representative the female and gestational age (week)-specific birthweight distributions from multiparous mothers.

The percentile charts (3rd, 5th, 10th, 50th, 90th, 95th and 97th centiles) were stratified by sex and mother’s parity. First of all, we showed smoothed percentiles for birthweight by gestational age (week) for male and female babies in Table 1. Then all infants from the primiparous mothers were grouped based on gestational age (week), and the data at the 3rd, 5th, 10th, 25th, 50th, 75th, 90th, 95th, and 97th percentiles were presented in Table 2. Infants from the multiparous mothers were plotted in the same way. The Table 3 showed smoothed percentiles for birthweight (in grams) of later-born male and female. As the gestational age (week) increases, the growth curves for various percentiles are smooth and increasing steadily. In the 10th, 50th, and 90th percentile graphs of singleton births, boys showed higher BWs than those of girls in the total infant graphs at each GA; moreover, Single births showed weight gained the most at 36–37 weeks, and growth slowed after 37 weeks (Fig. 1).

**Table 1:** Smoothed percentiles for birthweight (grams) of male and female.

GA (weeks)	Male babies smoothed percentiles											Female babies smoothed percentiles										
	*N*	*C3*	*C10*	*C25*	*C50*	*C75*	*C90*	*C97*	*L*	*M*	*S*	*N*	*C3*	*C10*	*C25*	*C50*	*C75*	*C90*	*C97*	*L*	*M*	*S*
25	64	555	632	700	772	848	926	1022	−0.26	772	326	23	635	705	768	834	903	972	1054	−0.23	834	153
26	228	634	715	787	862	940	1022	1124	−0.28	862	459	107	694	776	850	929	1011	1095	1197	−0.26	929	654
27	572	722	810	887	967	1052	1140	1251	−0.33	967	137	310	757	855	942	1035	1133	1236	1367	−0.35	1035	544
28	1273	810	911	1000	1092	1189	1291	1422	−0.37	1092	365	790	826	943	1047	1157	1275	1402	1571	0.14	1157	358
29	1698	889	1011	1117	1228	1344	1468	1630	−0.29	1228	289	1118	900	1040	1163	1293	1433	1589	1805	0.15	1293	408
30	2626	966	1110	1234	1363	1499	1646	1839	−0.28	1363	309	1670	981	1147	1289	1437	1599	1781	2037	0.09	1437	321
31	3795	1058	1223	1365	1512	1668	1835	2056	0.17	1512	305	2328	1087	1273	1433	1599	1777	1976	2253	0.16	1599	346
32	5605	1181	1366	1525	1689	1862	2047	2289	−0.13	1689	306	3750	1223	1427	1602	1784	1977	2186	2465	−0.15	1784	338
33	8513	1326	1528	1702	1882	2070	2270	2526	−0.08	1882	346	5726	1385	1601	1790	1986	2191	2408	2685	−0.10	1986	338
34	15824	1477	1695	1883	2078	2280	2492	2760	0.06	2078	336	11030	1569	1796	1995	2204	2420	2644	2920	0.08	2204	359
35	28548	1659	1888	2087	2294	2507	2728	3000	0.12	2294	360	20166	1768	2001	2208	2426	2651	2881	3158	0.11	2426	363
36	66292	1849	2085	2295	2513	2737	2966	3240	0.18	2513	367	46054	1980	2209	2418	2641	2872	3107	3387	0.13	2641	372
37	239695	2068	2303	2517	2744	2978	3213	3490	0.16	2744	366	165833	2214	2431	2635	2859	3094	3331	3609	0.16	2859	369
38	621082	2319	2540	2750	2979	3217	3455	3730	0.15	2979	367	474002	2399	2602	2800	3022	3257	3492	3762	0.14	3022	361
39	849913	2504	2713	2918	3147	3388	3627	3897	0.15	3147	372	751125	2510	2708	2907	3132	3372	3610	3878	0.33	3132	362
40	709857	2610	2818	3025	3260	3507	3750	4018	0.14	3260	383	688392	2564	2769	2976	3210	3458	3702	3974	0.30	3210	374
41	187571	2654	2870	3087	3332	3590	3841	4115	0.13	3332	392	197254	2634	2849	3064	3308	3565	3816	4093	0.28	3308	385
42	21317	2731	2954	3178	3431	3696	3953	4232	−0.28	3431	410	22022	2623	2846	3070	3324	3594	3858	4151	−0.23	3324	406

**Table 2:** Smoothed percentiles for birthweight (grams) of first born male and female.

GA (weeks)	First born male babies smoothed percentiles											First born female babies smoothed percentiles										
	*N*	*C3*	*C10*	*C25*	*C50*	*C75*	*C90*	*C97*	*L*	*M*	*S*	*N*	*C3*	*C10*	*C25*	*C50*	*C75*	*C90*	*C97*	*L*	*M*	*S*
25	12	657	725	788	856	926	993	1067	−0.26	856	326	6	600	673	738	805	872	936	1007	−0.27	805	798
26	58	721	803	878	958	1040	1120	1210	−0.28	958	459	28	667	753	830	910	990	1069	1157	−0.27	910	1252
27	138	792	892	982	1077	1174	1271	1383	−0.33	1077	137	80	732	833	923	1016	1112	1207	1318	−0.30	1016	1102
28	307	869	993	1101	1213	1327	1443	1584	−0.37	1213	365	198	803	920	1025	1135	1249	1366	1506	−0.39	1135	1161
29	440	953	1101	1227	1355	1487	1624	1796	−0.29	1355	289	297	883	1021	1144	1273	1408	1550	1725	−0.39	1273	1352
30	670	1047	1216	1358	1502	1650	1807	2008	−0.28	1502	309	448	968	1128	1270	1419	1577	1743	1955	−0.26	1419	1443
31	979	1162	1352	1511	1671	1838	2014	2243	0.17	1671	305	606	1065	1250	1413	1582	1760	1948	2189	0.27	1582	1602
32	1450	1300	1508	1684	1862	2046	2241	2490	−0.13	1862	306	1018	1190	1400	1581	1768	1962	2165	2420	0.06	1768	1764
33	2129	1461	1680	1869	2063	2262	2468	2726	−0.08	2063	346	1573	1351	1574	1766	1964	2167	2376	2633	−0.12	1964	1975
34	4195	1640	1867	2067	2273	2483	2697	2954	0.06	2273	336	3054	1547	1776	1976	2181	2391	2604	2859	−0.23	2181	2193
35	7699	1824	2058	2266	2482	2701	2919	3173	0.12	2482	360	5813	1752	1984	2189	2403	2620	2837	3092	−0.10	2403	2418
36	17663	2040	2272	2483	2704	2930	3152	3406	0.18	2704	367	12894	1956	2182	2387	2604	2826	3046	3302	0.19	2604	2614
37	59435	2274	2495	2702	2927	3159	3388	3648	0.16	2927	366	43413	2182	2394	2595	2813	3040	3266	3525	0.11	2813	2828
38	151706	2461	2668	2872	3099	3337	3572	3835	0.15	3099	367	122852	2368	2566	2761	2979	3209	3438	3697	0.25	2979	2996
39	222147	2579	2784	2990	3223	3469	3710	3975	0.15	3223	372	206784	2485	2680	2876	3098	3334	3567	3827	0.15	3098	3116
40	709857	2610	2818	3025	3260	3507	3750	4018	0.14	3260	383	187135	2547	2748	2951	3183	3428	3667	3930	0.14	3183	3199
41	187571	2654	2870	3087	3332	3590	3841	4115	0.13	3332	392	58249	2622	2829	3040	3281	3535	3780	4046	0.14	3281	3297
42	21317	2731	2954	3178	3431	3696	3953	4232	−0.28	3431	410	5434	2603	2817	3035	3286	3549	3803	4075	−0.20	3286	3297

**Table 3:** Smoothed percentiles for birthweight (grams) of later born male and female.

GA (weeks)	Later born male babies smoothed percentiles											Later born female babies smoothed percentiles										
	*N*	*C3*	*C10*	*C25*	*C50*	*C75*	*C90*	*C97*	*L*	*M*	*S*	*N*	*C3*	*C10*	*C25*	*C50*	*C75*	*C90*	*C97*	*L*	*M*	*S*
25	52	625	712	787	865	947	1035	1153	−0.36	865	211	17	619	685	747	814	885	959	1050	−0.20	814	146
26	170	722	814	892	974	1060	1153	1276	−0.38	974	316	79	688	769	843	923	1008	1098	1211	−0.33	923	499
27	434	817	919	1008	1100	1197	1303	1442	−0.33	1100	252	230	760	858	947	1042	1143	1252	1393	−0.23	1042	630
28	966	898	1019	1124	1233	1349	1475	1642	−0.35	1233	339	592	835	953	1058	1168	1288	1419	1594	−0.25	1168	394
29	1258	971	1113	1236	1365	1502	1650	1849	−0.30	1365	258	821	916	1054	1176	1304	1443	1598	1814	−0.23	1304	416
30	1956	1062	1226	1368	1517	1675	1847	2074	−0.25	1517	287	1222	1003	1165	1305	1452	1613	1795	2054	−0.23	1452	336
31	2816	1187	1370	1529	1696	1871	2059	2305	0.27	1696	288	1722	1105	1290	1450	1618	1800	2006	2297	0.21	1618	347
32	4155	1335	1535	1708	1888	2077	2278	2535	−0.23	1888	330	2732	1234	1438	1615	1802	2001	2221	2518	−0.12	1802	354
33	6384	1483	1701	1889	2084	2287	2500	2769	−0.18	2084	343	4153	1391	1607	1796	1995	2205	2428	2716	−0.33	1995	336
34	11629	1664	1894	2094	2302	2516	2739	3016	0.08	2302	353	7976	1575	1800	1999	2208	2426	2652	2931	0.11	2208	369
35	20849	1859	2096	2306	2524	2750	2981	3262	0.22	2524	380	14353	1774	2007	2214	2432	2659	2890	3169	0.16	2432	373
36	48629	2081	2317	2531	2759	2995	3234	3517	0.28	2759	383	33160	1991	2221	2430	2653	2885	3121	3404	0.36	2653	385
37	180260	2336	2557	2767	2996	3236	3476	3753	0.36	2996	376	122420	2231	2447	2651	2875	3110	3348	3627	0.27	2875	373
38	469376	2521	2730	2934	3163	3404	3643	3914	0.35	3163	371	351150	2412	2614	2812	3033	3269	3504	3772	0.25	3033	362
39	627766	2622	2831	3039	3273	3520	3763	4033	0.25	3273	376	544341	2519	2716	2914	3139	3379	3616	3883	0.24	3139	364
40	526358	2661	2878	3095	3341	3600	3852	4128	0.15	3341	392	501257	2568	2772	2978	3213	3461	3706	3978	0.09	3213	376
41	134437	2738	2962	3188	3443	3709	3968	4249	0.34	3443	403	139005	2633	2847	3063	3308	3566	3818	4094	0.24	3308	389
42	16498	2714	2949	3187	3457	3742	4018	4319	−0.25	3457	424	16588	2617	2843	3070	3327	3599	3865	4160	−0.17	3327	415

We use the Birth certificate data between Jan 1^st^, 2017 and Oct 31^st^, 2017 as our test dataset which were categorized into the 10^th^ and 90^th^ percentiles of birthweight for gestational age (week) (i.e., SGA, LGA) using cut points derived from our research standards, China’s 2015 research standards^13^ and INTERGROWTH-21st standards. The Table 4 provides the sex-specific proportions of these births at 25–42 gestational age (week) ranges.

**Table 4:** Comparison with the China’s 2015 Standard and INTERGROWTH-21st Standard.

GA (weeks)	*N*	Guangdong Standard			China’s 2015 Standard			INTERGROWTH-21st Standard		
		SGA(%)	AGA(%)	LGA(%)	SGA(%)	AGA(%)	LGA(%)	SGA(%)	AGA(%)	LGA(%)
25	22	13.64	81.82	4.55	0	90.91	9.09			
26	83	13.25	79.52	7.23	2.41	90.36	7.23			
27	229	12.66	82.53	4.80	2.62	93.89	3.49			
28	493	11.76	77.08	11.16	3.85	89.45	6.69			
29	691	11.14	78.00	10.85	3.47	90.45	6.08			
30	946	11.31	80.55	8.14	5.39	89.75	4.86			
31	1343	11.24	79.75	9.01	5.96	88.24	5.81			
32	2090	11.29	80.57	8.13	6.36	87.94	5.69			
33	3448	12.30	79.79	7.92	7.45	87.91	4.64	3.65	88.31	8.03
34	6562	10.06	81.04	8.90	7.99	87.11	4.91	5.61	86.64	7.76
35	11507	10.87	79.37	9.76	8.65	86.73	4.62	6.41	85.57	8.01
36	26147	11.01	79.97	9.01	8.57	86.71	4.72	6.02	85.15	8.84
37	93877	9.78	80.33	9.89	9.90	85.81	4.29	4.71	84.81	10.48
38	262138	10.67	78.49	10.84	11.10	84.50	4.40	5.10	85.27	9.62
39	376992	8.79	80.78	10.43	12.23	83.47	4.30	6.46	84.56	8.99
40	377739	11.43	79.76	8.80	16.12	79.53	4.35	11.61	81.58	6.82
41	75187	9.55	80.69	9.76	13.72	80.07	6.21	11.96	81.66	6.38
42	5870	10.51	79.98	9.51	16.75	76.78	6.47	19.01	76.15	4.84
total	1245364	10.21	79.91	9.88	12.93	82.59	4.48	7.98	83.65	8.37

**Figure 2:**
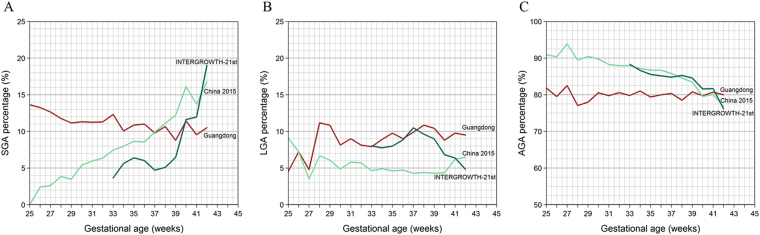
Comparison with the China 2015 Standard and INTERGROWTH-21st Standard. (**A**) In each gestational age (week), the SGA rate is calculated by dividing the number of infants who are judged as SGA by the total number of infants born during this gestational age (week). The number of AGA infants were determined by three criteria. (**B**) The LGA rate is calculated by dividing the number of infants who are judged as LGA by the total number of infants born during this gestational age (week). The number of LGA infants were determined by three criteria. (**C**) The AGA rate is calculated by dividing the number of infants who are judged as AGA by the total number of infants born during this gestational age (week). The number of AGA infants were determined by three criteria.

The curves show the incidence of SGA at each gestational age (week), and the three criteria are compared (Fig. 2). In the same way, the incidence of LGA and the incidence of AGA in each gestational age (week) was observed by these three criteria. As the INTERGROWTH-21st national standards only cover 33–42 weeks of gestational age (week), we only calculate the 10th and 90th percentiles of birthweight for gestational age (week) (i.e., SGA, LGA) at 33–42 weeks using this reference. As expected, the thresholds derived from INTERGROWTH-21st standards below the 10th and above the 90th percentile across all gestational age (week) categories were from 3.65% to 19.01%. On the other hand, the thresholds derived from China’s 2015 research standards captured a greater proportion of SGA births (16.75% in 42 gestational age (week), while included only 9.09% (25 gestational age (week)) of LGA births within the gestation ranges in their research dataset. In our research, the 10th and 90th-percentile proportions of birthweight for gestational age (week) were relatively stable. The maximum value was found in SGA of 25 gestational age (week) (13.64%), while the minimum value is found in LGA of 25 gestational age (week) (4.55%). The overall prevalence of SGA estimated by our standards, the China’s 2015 research standards and INTERGROWTH-21st standards, were 10.21%,12.93% and 7.98%, respectively, whereas the overall prevalence of LGA was 9.88%, 4.48% and 8.37%, respectively.

## Discussion

The birthweight references included newborns with adverse pregnancy outcomes. These references served the purpose of comparing newborns with the general population, but they were not the prescribed criteria on how newborns should grow under the optimal pregnancy conditions. Infant birthweight is influenced by both environmental and genetic factors; therefore, it is important to identify the percentile distribution of birthweight during pregnancy using recent data to evaluate infants^16^. The birthweight standard for Chinese infants was published in 1988, which was nearly three-decade old and cannot reflect the current newborns’ situation^17^. The male birthweight is bigger than that of female; thus, the birthweight curve should be sex-specific. For example, a cross-sectional population-based study in Australia between 1998 and 2007, by comparing term babies of the same gestational age (week), the median birthweight is 0–25 g heavier for male infants, and 5–45 g higher for female infants than 10 years ago^11^. In our analyses, from 25 gestational age (week) to 42 gestational age (week), the average birthweight of males is from 903 g to 3470 g. The average birthweight of females is from 844 g to 3341 g. The 3rd, 5th, 10th, 50th, 90th, 95th and 97th centiles birthweight of males is heavier than that of girls. In 1988, Zhang *et al*.^18^ reported the physical development of different gestational age (week) neonates in 15 cities in China. Their study showed that from 28 gestational age (week) to 42 gestational age (week), the birthweight of the male neonate from 50th were from 1234 g to 3405 g. The 50th birthweight of female newborns were from 1103 g to 3292 g. In 2015, a nationwide neonatology network in China made a survey in 63 hospitals. And the mean birthweight of male was (3271 ± 576) g, the mean birthweight of female was (3188 ± 528) g^13^. Compared with references using previously published percentiles in Australia, increases in age-specific 10th and 90th percentiles observed from current data will therefore increase the rate of SGA and decrease the rate of LGA for term births^11^. In the United States, the 50th percentile birthweight of male and female at 40 gestational age (week) were respectively 3572 g and 3431 g in 2011^9^, which were higher than those published in China’s 2015 research birthweight standard (3482 g for male and 3349 g for female). In Guangdong population, the birthweight at 50 percentiles was 3339 g and 3213 g for male and female respectively. In general, birthweight in China is smaller compared to that in the developed countries, while the BWs in Guangdong province is even smaller than China’s average.

The average birthweight of primipara male infants at 40 gestational age (week) was 120 g higher than that of female. The largest mean birthweights gap between the male and the female infants was 135 g at 41 gestational age (week). These gender differences are bigger than the ones in India where the term firstborn males were found to be 45 g heavier than females on average (the mean birthweight were 2934 g and 2889.5 g for males and females respectively). When considering later born preterm babies, the males outweighed the female babies by 111 grams. The mean birthweight were 2089 g and 1978 g respectively^19^.

In Australia, a mean increase in birthweight of 23 g from 1990 to 2005 for male babies in New South Wales could be translated into an 18% increase in those identified as SGA, or 21% increase in those identified as LGA for females. The test data from 2017 shows between 25 and 27-week gestational age (week), we observed the highest prevalence (12.66~13.64%) of SGA by applying the new Guangdong Province birthweight standard. In contrast, by applying the same standard, the incidence of SGA was the lowest (8.79%) in 39 gestational age (week). Relative to the other two standards, the prevalence of LGA was the highest estimated by our research standards in general. The use of the other two percentiles may lead to the unnecessary intervention and anxiety to the parents of babies whose weight fall in the range of lower and upper centiles according to our centiles. With the China’s 2015 research standards, the total incidence of SGA is the largest (12.93%), while the total incidence of SGA by our research and International standards is 10.21% and 7.98% respectively. On the contrary, the total incidence of LGA by the 2015 China standard is lowest (4.48%) compared to the other two standards.

A study has shown that infants defined by Guangdong new birthweight reference (a new reference) as SGA, 15.3–47.7% (depending upon gestational age (week)) were considered appropriate for gestational age (week) (AGA) by the currently used reference of China. Of the infants defined as SGA by the new reference, 92% with gestational age (week) between 34 and 36 weeks and 14.3% between 37 and 41 weeks were considered AGA by the global reference^20^.

Our method to assess gestational age (week) may be constrained by methodology. Ideally, gestational age (week) should be combined with the assessment of mother’s last menstrual period, prenatal ultrasound measurement, and postnatal assessment; such practice could be found from the National Perinatal Data Collection (NPDC) of the Australian Institute of Health and Welfare (AIHW) National Perinatal Statistics Unit^11,21^ and Scottish neonatal birthweight percentiles by Bonellie *et al*.^22^. However, in actual clinical practice in Guangdong, the gestational age (week) assessment was mainly assessed by mother’s last menstrual period. If the conditions of midwifery technical services of medical institutions allow, the early pregnancy ultrasound correction of gestational age (week) may have been carried out. Although the ultrasound examination to assess the gestational age (week) has been commonly used, and the regular pregnancy test performed in the early gestational age (week) also becomes popular, it is difficult to collect obstetric information; and therefore, most of the newborn’s gestational age (week)s are still solely based on mother’s last menstrual time.
